# Supplementary material for: Urban density and spatial planning: The unforeseen impacts of Dutch devolution
Source: PLoS One. 2020 Oct 15;15(10):e0240738. doi: 10.1371/journal.pone.0240738 (PMC7561180; doi:10.1371/journal.pone.0240738)
Supplement: S1 Table — (DOCX) [file pone.0240738.s001.docx]

S1 Table: Aggregation of land-use classes

| Original Land-use class | Aggregated Land-use class | Development Process |
| --- | --- | --- |
| Railways | Infrastructure | Greyfield |
| Main roads | Infrastructure | Greyfield |
| Airport | Infrastructure | Greyfield |
| Residential | Residential | Residential |
| Retail and hospitality/catering | Other built-up | Greyfield |
| Public facilities | Other built-up | Greyfield |
| Social-cultural facilities | Other built-up | Greyfield |
| Industrial grounds | Other built-up | Greyfield |
| Dumping grounds | Other built-up | Greyfield |
| Wreck storage facilities | Other built-up | Greyfield |
| Cemetery | Other built-up | Greyfield |
| Mining | Other built-up | Greyfield |
| Construction sites | Construction | Greyfield |
| Semi-paved terrain | Other built-up | Greyfield |
| Parks | Urban Green | Green |
| Sports facilities | Urban Green | Green |
| Allotment gardens | Urban Green | Green |
| Day trip locations | Other built-up | Greyfield |
| Accommodations | Other built-up | Greyfield |
| Greenhouses | Agriculture | Green |
| Other agricultural | Agriculture | Green |
| Forest | Nature | Green |
| Dry natural area | Nature | Green |
| Wetlands | Nature | Green |
| IJsselmeer/Markermeer | Water | Green |
| Closed estuary | Water | Green |
| Rhine and Meuse | Water | Green |
| Randmeer | Water | Green |
| Reservoir | Water | Green |
| Water with recreational function | Water | Green |
| Water with extraction function | Water | Green |
| Mud depot | Other built-up | Greyfield |
| Other inland water | Water | Green |
| Waddenzee, Eems, Dollard | Exterior water | Green |
| Eastern Schelde estuary | Water | Green |
| Western Schelde estuary | Water | Green |
| North Sea | Exterior water | Green |
| Foreign country | Outside Study Area | - |
