# Supplementary material for: Urban density and spatial planning: The unforeseen impacts of Dutch devolution
Source: PLoS One. 2020 Oct 15;15(10):e0240738. doi: 10.1371/journal.pone.0240738 (PMC7561180; doi:10.1371/journal.pone.0240738)
Supplement: S2 Table — (DOCX) [file pone.0240738.s002.docx]

S2 Table: Actual development values per period for the three main development processes

| Urban residential densification | Positive | Negative | Saldi |
| --- | --- | --- | --- |
| 2000-2006 | 129,740 | -49,840 | 79,900 |
| 2006-2012 | 135,680 | -61,570 | 74,110 |
| 2012-2018 | 137,057 | -48,696 | 88,361 |
|  |  |  |  |
| Urban greyfield redevelopment |  |  |  |
| 2000-2006 | 51,715 | -7,520 | 44,195 |
| 2006-2012 | 78,135 | -9,930 | 68,205 |
| 2012-2018 | 127,857 | -15,857 | 112,000 |
|  |  |  |  |
| Non-urban greenfield development |  |  |  |
| 2000-2006 | 144,905 | -3,670 | 141,235 |
| 2006-2012 | 166,085 | -6,000 | 160,085 |
| 2012-2018 | 103,969 | -5,938 | 98,031 |
