# Supplementary material for: Urban density and spatial planning: The unforeseen impacts of Dutch devolution
Source: PLoS One. 2020 Oct 15;15(10):e0240738. doi: 10.1371/journal.pone.0240738 (PMC7561180; doi:10.1371/journal.pone.0240738)
Supplement: Attachment — Submitted filename: Reviewer_PONE-D-20-22192.docx [file pone.0240738.s003.docx]

Reviewer’s Report

Title: Urban density and spatial planning: the unforeseen impacts of Dutch devolution

Reviewer: Ebenezer N. K. Boateng

Reviewer's report:

Thank you for the opportunity to review the manuscript entitled “Urban density and spatial planning: the unforeseen impacts of Dutch devolution”. The authors investigated residential development since 2000 in the Netherlands in relation to the changing planning policies. The study found that while the national policy on residential development is decentralised making it look weak and the share of residential development within existing urban areas increased. The findings of this study contribute to literature especially its methodological approach. In general, this is a well-written manuscript. However, I have a few suggestions that when considered can help improve the manuscript.

**Topic**

1. The topic appears great because it is clear and specific to the purpose of the study.

**Abstract**

2. The sentences should be in the past. For instance, page 2 line 17 and 19 should be **we focused**… and **we quantified**… respectively.
3. Keywords: Please add rural women

**Background**

4. **T**he background and problem statement looks great but kindly state the specific objectives.
5. Kindly add the significance of the study to the background.

**Dutch Context**

6. This section was well explained

**Methods**

7. The study design was not too clearly stated.
8. Please check the sentence of page 7 line 150, it should read …, **we focused on the**…
9. Is there any justification for putting brownfield redevelopment into bracket (line 177)?
10. Please check the sentence of line 181, it is supposed to read **we use**

**Results and discussions**

11. The results presented were great but the conclusion made could it be all summed in the conclusion and recommendation? If not, kindly state it as your key findings. For instance, line 300 *“This is our first important* ***conclusion****: the disappearance of national coordination and the abolition of development subsidies did not result in a drop in the number of housing units added to the stock”.* Similar of such conclusion could be found in line 309.
12. In some of the interpretations, please refer to the exact table number. For instance, line 303, “*The table (****Table 2****) shows a large reduction in the share of new housing constructed outside existing urban areas: from 58% in the first period to 31% in the third*”. This is similar in the following lines; line 323 and 373.
13. In line 372, the **saldi** stated here cannot be found in table 2. Kindly check.
14. Well synthesised, Good job done!

**Conclusion and recommendation**

15. This was done well but line 466-467 talks about proximity to certain amenities was not mentioned in the results and discussion so how do you draw conclusion on that?
