# Supplementary material for: Urban density and spatial planning: The unforeseen impacts of Dutch devolution
Source: PLoS One. 2020 Oct 15;15(10):e0240738. doi: 10.1371/journal.pone.0240738 (PMC7561180; doi:10.1371/journal.pone.0240738)
Supplement: Attachment — Submitted filename: Repliek voor reviewers.docx [file pone.0240738.s004.docx]

**Comments from the editors**

*When submitting your revision, we need you to address these additional requirements.*

*1. Please ensure that your manuscript meets PLOS ONE's style requirements, including those for file naming.*

We have again checked the formatting requirements and changed the capitalisation of the title, and the style of the headings. We assume that we now conform to the style requirements of this journal.

*2. Thank you for stating the following in the Acknowledgments Section of your manuscript: "This research was financially supported by the Dutch Research Council (NWO) as a SURF Pop up project." We note that you have provided funding information that is not currently declared in your Funding Statement. However, funding information should not appear in the Acknowledgments section or other areas of your manuscript. We will only publish funding information present in the Funding Statement section of the online submission form. Please remove any funding-related text from the manuscript and let us know how you would like to update your Funding Statement. Currently, your Funding Statement reads as follows: "The author(s) received no specific funding for this work."*

We have now omitted the funding information from the acknowledgements and included it in the Funding Statement section of the submission form.

**Reviewer #1**

*Thank you for the opportunity to review the manuscript entitled “Urban density and spatial planning: the unforeseen impacts of Dutch devolution”. The authors investigated residential development since 2000 in the Netherlands in relation to the changing planning policies. The study found that while the national policy on residential development is decentralised making it look weak and the share of residential development within existing urban areas increased. The findings of this study contribute to literature especially its methodological approach. In general, this is a well-written manuscript. However, I have a few suggestions that when considered can help improve the manuscript.*

Thanks for your kind comments.

*Topic*

*1. The topic appears great because it is clear and specific to the purpose of the study.*

Thanks again.

*Abstract*

*2. The sentences should be in the past. For instance, page 2 line 17 and 19 should be we focused… and we quantified… respectively.*

We have changed the tense in the abstract and entire method section for consistency.

*3. Keywords: Please add rural women*

In relation to rural development we have listed the keyword greenfield redevelopment. The gender aspect of these developments is not discussed, so we have note included this suggestion.

*Background*

*4. The background and problem statement looks great but kindly state the specific objectives.*

We have now emphasised the objectives in the introduction.

*5. Kindly add the significance of the study to the background.*

We have formulated the significance of the study in a more explicit way in the introduction section.

*Dutch Context*

*6. This section was well explained*

Thanks for this positive comment.

*Methods*

*7. The study design was not too clearly stated.*

We now sketch the basic study design at the start of the Methods section.

*8. Please check the sentence of page 7 line 150, it should read …, we focused on the…*

We have changed the tense throughout this section.

*9. Is there any justification for putting brownfield redevelopment into bracket (line 177)?*

In this research brownfield redevelopment is combined with greyfield redevelopment because of a lack of appropriate data, that has now been clarified in the caption of Fig 1 and the main text.

*10. Please check the sentence of line 181, it is supposed to read we use*

The sentence has been adapted to the past tense and now reads “we used”.

*Results and discussions*

*11. The results presented were great but the conclusion made could it be all summed in the conclusion and recommendation? If not, kindly state it as your key findings. For instance, line 300 “This is our first important conclusion: the disappearance of national coordination and the abolition of development subsidies did not result in a drop in the number of housing units added to the stock”. Similar of such conclusion could be found in line 309.*

We have included more explicit conclusions to the results section and conclusion.

*12. In some of the interpretations, please refer to the exact table number. For instance, line 303, “The table (Table 2) shows a large reduction in the share of new housing constructed outside existing urban areas: from 58% in the first period to 31% in the third”. This is similar in the following lines; line 323 and 373.*

We have added the explicit table number in this sentence and also to two other instances in the Results section.

*13. In line 372, the saldi stated here cannot be found in table 2. Kindly check.*

We have described more explicitly now why the saldi here differ from Table 2.

*14. Well synthesised, Good job done!*

Thanks again for your kind words.

*Conclusion and recommendation*

*15. This was done well but line 466-467 talks about proximity to certain amenities was not mentioned in the results and discussion so how do you draw conclusion on that?*

We now more clearly indicated that the urban attractivity index represents a neighbourhood related amount of amenities.

**Reviewer #2**

*The manuscript is technically strong and is a good scientific research with data that supports the conclusions. The conclusions is appropriately based on the data presented and the statistical analysis has been performed appropriately and the manuscript presented in an standard English. I would like to be able to offer more precise data on the evolution of housing prices and to establish clearer recommendations for public policy.*

Thank you for this very positive review. In response we have now included a precise figure related to house price development in Section 4.1.

In the conclusion we now introduced more specific public policy recommendations.
